# Consensus on key domains for emergency medical teams deployment evaluation: a Delphi method study

**DOI:** 10.1186/s13031-026-00751-y

**Published:** 2026-02-05

**Authors:** Tiffany Yeung, Daniel G. Bausch, Arlinda Cerga Pashoja, Joanna Schellenberg

**Affiliations:** 1 Department of Disease Control, London School of Hygiene & Tropical Medicine, Keppel Street, London, WC1E 7HT UK; 2 Centre for Infectious Disease Emergency Response, National University of Singapore, 21 Lower Kent Ridge Rd, 119077 Singapore, Singapore; 3 Faculty of Sport, Technology and Health Sciences, St Mary’s University, Waldegrave Road, Strawberry Hill, Twickenham, TW1 4SX UK

**Keywords:** Natural disasters, Humanitarian, Emergencies, Disaster aid, Evaluation, Delphi method

## Abstract

**Background:**

Emergency Medical Teams (EMT) deploy to provide urgent care during and after sudden onset disasters. Although the World Health Organisation has set standards for EMTs on training, personnel, and operations, there is presently no standardised evaluation framework to assess deployments.

**Methods:**

As a step towards creation of an EMT deployment evaluation framework with an agreed upon common set of criteria, we used a two-round modified Delphi method to elicit perspectives from key stakeholders, including EMT members, researchers, funders, representatives of deploying governments, and host organisations. We asked participants to rate themes and questions relevant to EMT evaluation, derived from a previous study, using a four-point Likert scale, with consensus considered reached when 75% or more respondents rated the themes or questions ‘slightly suitable’ or ‘highly suitable’.

**Results:**

Two rounds of the Delphi process were completed by 15 and 16 participants, respectively, with all first-round participants plus an additional person participating in the second round. Participants reached consensus on all 23 proposed themes and 162 of 165 (98%) proposed questions, with near unanimous views on many: 17 themes and 55 questions reached 100% consensus.

**Conclusions:**

Based on the consensus findings and guided by the pillar themes and questions outlined in the World Health Organization’s After Action Review guidance, we proposed a preliminary structure for an EMT evaluation. These findings can serve as a foundation to inform the future design of a standardised evaluation framework. Further refinement through iterative testing and stakeholder consultation will enhance the framework’s practicality and adaptability.

**Supplementary Information:**

The online version contains supplementary material available at 10.1186/s13031-026-00751-y.

## Background

An Emergency Medical Team (EMT) is composed of health professionals providing urgent care during sudden onset disasters, both “natural” and human-caused [1]. In 2014, the World Health Organization (WHO) launched the EMT Initiative to set common standards for EMT healthcare services to enhance and ensure response quality. The EMT initiative includes a process of EMT classification, encouraging all EMTs to register within the WHO EMT system, and have their services and skills validated by the initiative [2].

Initially focused on surgical care following earthquakes, the WHO EMT Initiative has adapted to new challenges, creating guidelines for infectious diseases and conflict zones. While the WHO EMT initiative sets minimum standards, countries may nevertheless accept teams that have not been classified by WHO, leading to concerns about EMT service quality and accountability. Even before the WHO initiative was established, there were concerns regarding timing, relevance, and integration of EMT activities with local health systems [3–6]. Scepticism regarding the motivations behind international aid continues to grow, particularly among beneficiaries, and media critiques often hold EMTs accountable more effectively than funders [7, 8]. Transparency in evaluation methods is critical in learning from past deployments [9]. Recent literature stresses the need for quality assurance and adherence to WHO standards [10, 11].

Despite a 2023 study highlighting the need for standardised reporting and sharing of experiences [12], the EMT sector lacks a standardised evaluation framework to assess EMT deployments. Instead, it often relies on daily reports submitted during deployment that emphasise clinical data, an approach that is not conducive to overall assessment of accountability and effectiveness and making it hard to draw lessons across deployments [7, 11, 13, 14]. EMT evaluation that have been performed tend to focus on funder requirements rather than improvements. There is often reluctance to share data or publish data [15, 16], perhaps to avoid manifesting shortcomings in expectations and performance gaps [6, 17–19], rendering it difficult to make evidence-based decisions [7, 11, 13, 14, 20]. The publications that do exist often focus on descriptive narratives and ‘lives saved’, rather than a broader range of measures that can provide a better idea on usage of resources to better support local health infrastructure [13, 18, 19, 21–23]. Standardised evaluations of EMT deployments could help improve adherence to the standards set by WHO [3, 24]. In its strategic priorities for 2022–2024, the Inter-Agency Standing Committee, an inter-agency forum of the United Nations and non-UN humanitarian partners founded in 1991 to strengthen humanitarian assistance, included improving ‘Accountability to Affected People,’ ensuring that no one is overlooked, and that community feedback is considered [25, 26]. However, international disaster rules and standards require improved implementation, and practical methods to support adherence must be developed.

While there are no established frameworks for EMT evaluations, various prominent organisations in the humanitarian field have developed frameworks for longer-term humanitarian assistance. An extensive review of existing reports, documents, and guidelines related to humanitarian assistance evaluations we did reveals that, although most humanitarian organisations have their own evaluation frameworks, the criteria used vary widely, and are generally not directly applicable to EMT evaluations, which require quicker assessments during EMT deployments compared to the longer humanitarian assistance [27].

Given the lack of an evaluation framework for EMT deployments, we sought to gather views of experts in this field on key themes and questions to be incorporated into a future EMT evaluation, with a goal of eventually producing a framework that can demonstrate accountability, foster discussions for improvement and knowledge sharing, and provide an overview of resource allocation. We focussed on the three most common types of disasters that required international aid: floods, tropical cyclones, and earthquakes and related tsunamis.

The Organisation for Economic Co-operation and Development classifies evaluations into five types: formative, process, output, outcome or performance, and impact [28]. This study focussed on process, output, and outcome evaluations, which assess short-term outputs, which are most relevant to EMT deployments.

## Methods

The reporting in this paper adheres to the Conducting and Reporting DElphi Studies (CREDES) checklist [29].

### Study design

This is a mixed methods study using the Delphi method, including both qualitative and quantitative data.

### Ethics approval

Ethics approval was provided by the London School of Hygiene & Tropical Medicine Ethics Committee (Ref: 29517). Participants gave written informed consent before each round of the Delphi questionnaire.

### The Delphi method

**Fig. 1:**
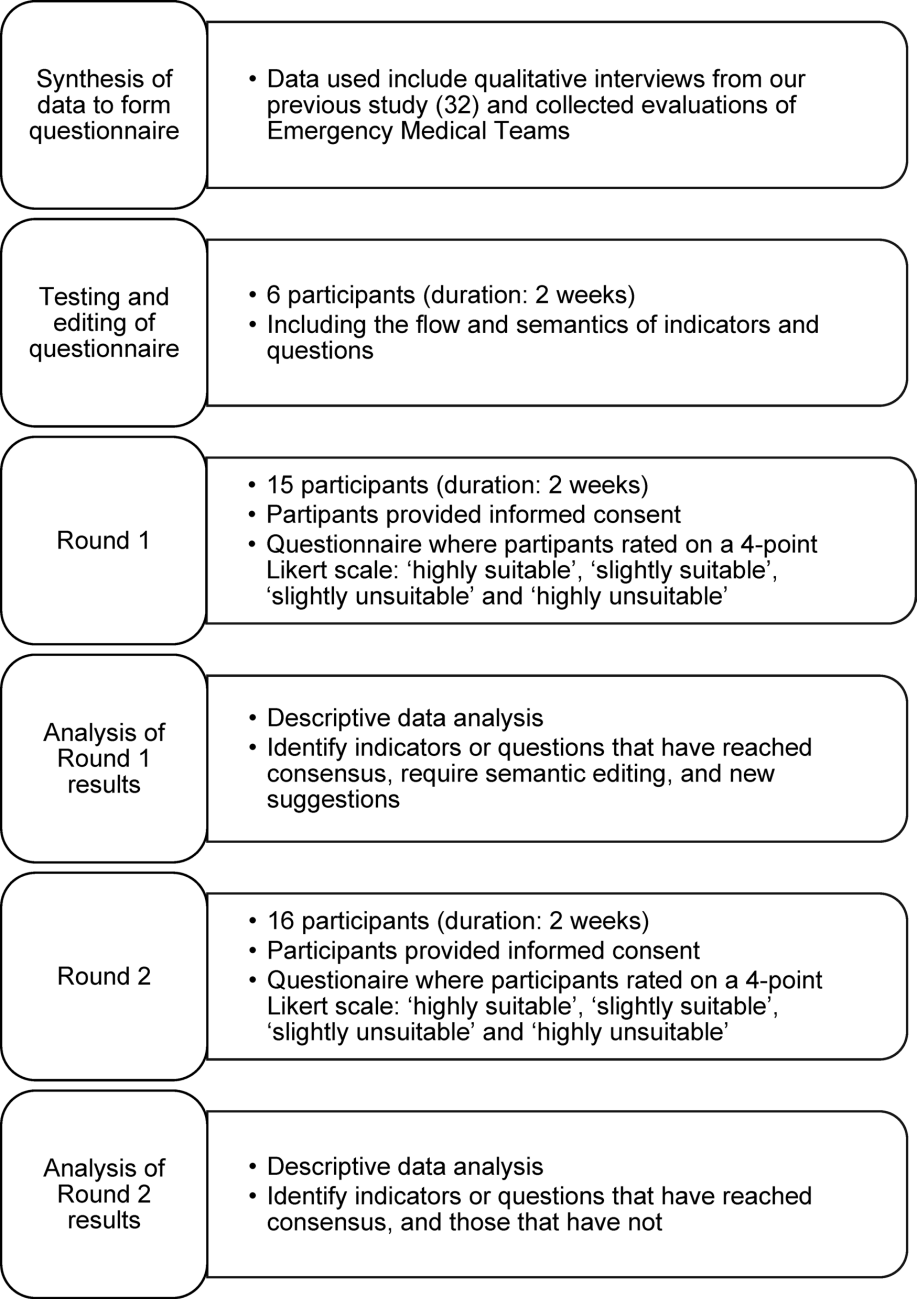
Stages of the Delphi Method

The Delphi method is a widely used approach that facilitates consensus among experts, particularly in the development of standards, frameworks, and guidelines [30]. We selected the Delphi method to obtain expert consensus on a relatively novel topic. This approach allows participants to remain anonymous, thereby reducing the influence of dominant individuals and facilitating unbiased consensus-building. We employed a modified Delphi method, drawing on findings from published literature and qualitative interviews to inform the development of the first-round questionnaire [31]. As a result, the process did not begin with open-ended questions [32]. We administered two rounds of questionnaires. In addition to questions about the content of an EMT evaluation, in Round 1 we included questions related to the process – who should conduct the evaluation and how long post-EMT deployment. Figure 1 shows the stages of the Delphi method process in this research, from the preparation to analysis.

In a previous study we generated common evaluation themes and questions from interviewing EMT stakeholders about their perspectives and current practices in evaluating EMT deployments [31]. Responses to interview questions regarding indicators of successful EMT deployments, necessary data for improving future operations, and participants’ prior experiences and evaluative ideals yielded a set of variables critical to deployment assessment, which we subsequently rewrote as questions, grouped thematically, and organised within the six After Action Review pillars established by the WHO to assess response to significant public health events (Table 1) [33].

**Table 1:** Evaluation themes that reached consensus across two rounds of Delphi method

After Action Review Pillar	Evaluation themes	Agreement Intensity (Absolutely Suitable + Slightly Suitable)
1. Leadership	Leadership	100%
	Teamwork	100%
2. Partner coordination	Deploying organisation	100%
	Local Communities	93%
	Host Organisations	100%
	World Health Organization, Emergency Medical Teams Coordination Cell and other Emergency Medical Teams	100%
3. Information management and planning	Preparation of evaluation	93%
	Information Management (new in Round 2)	100%
	Documentation	100%
4. Health operations and technical expertise	Team Members’ Experience	100%
	Filling in gaps of needs	86%
	Patient Numbers	100%
	Clinical Care	100%
	Referral of Patients	100%
	Local engagement	100%
	Post-deployment follow-up	100%
5. Operations support and logistics	Arrival	100%
	Quality assurance	100%
	Equipment, supplies and logistics	93%
	Safety (new in Round 2)	94%
	Exit	100%
6. Finance and administration	Finance & Administration	100%
	Publications and reports	100%

The After Action Review was developed as part of the WHO International Health Regulations Monitoring and Evaluation Framework to evaluate nation-wide emergency response but is not specifically oriented toward individual EMT’s actions. Rather, its pillars represent broad, overarching areas, from which WHO suggests that users design an appropriate review framework specific to each response based on the different contexts. Thus, while it served as a useful framework for development of questions and themes for this study, the WHO After Action Review Toolkit cannot serve in and of itself as an EMT evaluation.

We asked participants to rate the evaluation themes and respective questions on their perceived suitability for an EMT deployment evaluation framework, using a four-point Likert scale: ‘highly suitable’, ‘slightly suitable’, ‘slightly unsuitable’ and ‘highly unsuitable’. We then gave participants the opportunity to explain their choices, as well as to provide new suggestions to be added to the list.

Round 2 included evaluation themes and questions that had not reached consensus in the first round, along with items that received suggestions for modification and new proposals emerging from participants’ comments in Round 1. For questions that reached consensus but were accompanied by suggested revisions, we asked participants to indicate whether they preferred the original version or supported the proposed changes.

We considered all participant comments provided in both rounds of the Delphi, adjusting and incorporating suggestions for new evaluation themes and questions from Round 1 into Round 2 as appropriate. We took note of any patterns to identify respondents who consistently provided low ratings.

### Sampling and recruitment

A panel of 10–15 experts is considered ideal for the Delphi method [34]. Through purposeful sampling based on their roles and experience working in or with EMTs, we identified and sent invitations to 27 potential participants representing various stakeholder groups, accounting for potential non-responses (Table 2). The inclusion criteria required participants to hold a leadership role and have experience working with or within EMTs during floods, tropical cyclones, or earthquakes and related tsunamis, both before and after the COVID-19 pandemic.

**Table 2:** Characteristics of participants in the Delphi study

Parameter	Participants, *n* (%)	
	Round 1	Round 2
Type of stakeholder		
Researchers in Emergency Medical Teams and humanitarian assistance	3 (20%)	3 (19%)
World Health Organization classified and unclassified Emergency Medical Teams	8 (53%)	9 (56%)
Deploying governments or funders	1 (7%)	1 (6%)
Host governments	2 (13%)	2 (13%)
Host partners (e.g., local health services)	1 (7%)	1 (6%)
Gender		
Male	11 (73%)	11 (69%)
Female	4 (27%)	5 (31%)
World Bank income groups of participants’ nationality		
Low income	0 (0%)	0 (0%)
Lower-middle income	1 (7%)	1 (6%)
Upper-middle income	5 (33%)	5 (31%)
High income	9 (60%)	10 (63%)
World Health Organisation Region of participants’ nationality		
Africa	0 (0%)	0 (0%)
Americas	1 (7%)	2 (13%)
Eastern Mediterranean	2 (13%)	2 (13%)
Europe	7 (47%)	7 (44%)
South-East Asia	1 (7%)	1 (6%)
Western Pacific	3 (20%)	3 (19%)

### Piloting the questionnaire

We piloted the first Delphi questionnaire with seven individuals, five with substantial experience working in EMTs and two with expertise in questionnaire administration who provided feedback on structure and flow. These individuals and their responses were not included in the subsequent study. Based on feedback from the pilot, we revised the questionnaire layout, introductory text, and wording to enhance clarity. We used an online surveying tool, Jisc Online Surveys (Jisc, United Kingdom). The questionnaire was available in English only.

### Data analysis

For each round, we defined consensus as 75% or more participants rating the evaluation themes and questions as ‘slightly suitable’ or ‘highly suitable’. Questions that reached consensus but did not yield agreement on suggested modifications were retained within the ‘reached consensus’ category, with annotations indicating the lack of consensus on the proposed changes. Similarly, for suggested changes, we considered the change agreed if 75% or more participants rated ‘I agree with the change’.

## Results

### Participants

Of the 27 invited participants, 15 (56%) participated in Round 1 and 16 (59%) in Round 2 (Table 2). All Round 1 participants also took part in Round 2, with one additional participant joining in the second round. The remaining invited individuals who did not participate offered no explanations for their lack of participation.

### Delphi method findings

**Fig. 2:**
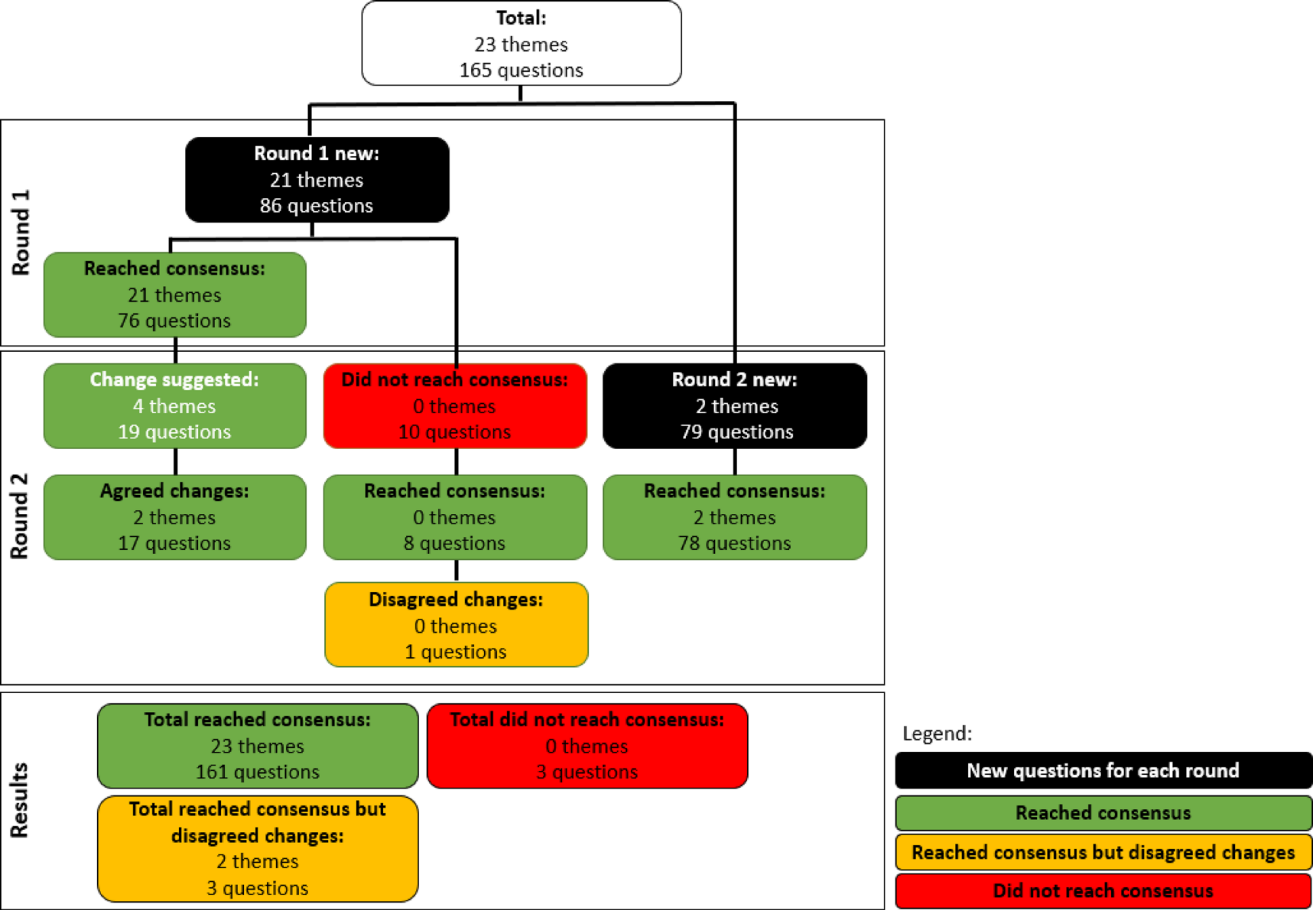
The Flow of Evaluation Themes and Questions Over Two Delphi Rounds

After two rounds of the Delphi process, all 23 proposed evaluation themes and 162 out of 165 questions achieved consensus (Fig. 2). Consensus was unanimous (100%) for 17 themes and 55 questions (Table 1). Notably, the majority of items receiving 100% consensus fell under the After Action Review pillar of “Health operations and technical expertise”, suggesting that this theme consistently aligns with the views and interests of participants. Many themes and questions also aligned closely with the existing literature on EMTs, indicating their prominence in current EMT publications. A full list of the evaluation themes and questions and their respective level of agreement is contained in Appendix 1.

For the three themes and questions on which consensus was reached but without agreement on proposed changes, the disagreements were largely related to syntax (i.e. “host organisation” versus “local government” or “ministry of health”) and how a particular theme (“Preparation of evaluation”) should be categorised (i.e. under “Information management and planning” or “Operations support and logistics”).

Consensus was not reached for three questions:

1) How long did it take for patients to arrive at the hospital? (Agreement *≤* 69%. No comments provided.)

2) Did the EMT do any follow-up to patients after the it exited the country? (Agreement *≤* 63%. Five participants commented on the difficulty and infeasibility of doing this in the field.)

3) Were there repeat visits of EMT members to the disaster area after the deployment? (Agreement *≤* 69%. No comments provided.)

### Conducting the evaluation

**Fig. 3:**
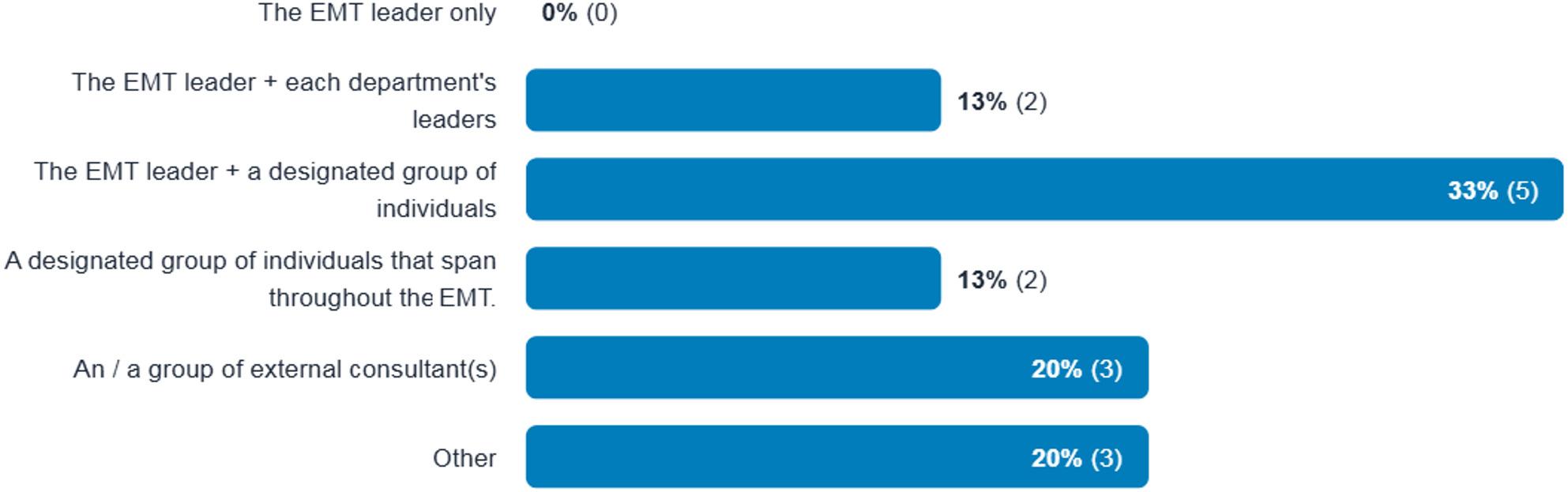
Participants' Response on Who Should be Involved in the Evaluation of an EMT Deployment

Participants’ views on who should conduct evaluations varied, but most thought it required a team approach, often including the EMT leader, and sometimes external consultants (Fig. 3).

**Fig. 4:**
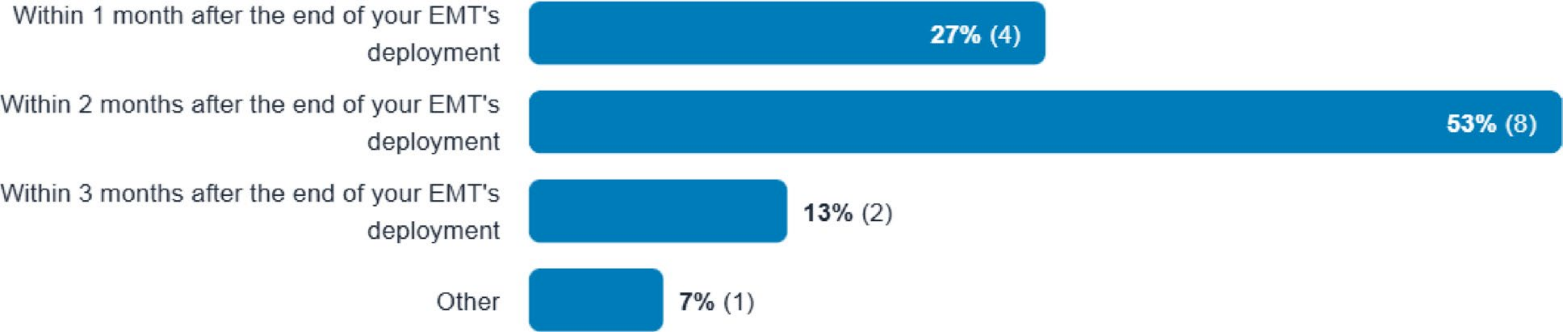
Participants’ Response on When Should the Evaluation be Completed

A majority (53%) of participants thought that the evaluation should take place within two months after end of the deployment, and 93% thought within three months (Fig. 4).

## Discussion

Despite widespread agreement of the need, there is currently no standardised framework for evaluating EMT deployments. Our study represents a first step toward a solution, highlighting relevant themes and questions for such a framework for which there is significant consensus, indeed nearly unanimity, among key stakeholders.

The Delphi method proved to be a useful tool, ensuring anonymity and preventing “groupthink”, which can lead to unchallenged, poor-quality decision-making [35]. In the two-round Delphi method, consensus was achieved on all 23 evaluation themes and 162 (98%) of 165 questions for EMT deployments, including unanimous (100%) agreement on 17 themes and 55 questions. Many of these themes and questions were previously noted in the existing literature. Minor disagreements generally pertained to syntax, such as “host organisation” versus “local government”, or the positioning of a particular theme within the document. For questions that reached consensus but did not reach agreement, standard terms used in WHO’s Classification and Minimum Standards for EMTs, or the After Action Review guideline, could be used to guide the choice of words in future studies and a final EMT evaluation framework. The overwhelming majority (93%) of participants recommended that evaluation occur within three months post-deployment, rejecting evaluation of themes or questions that would require longer-term follow-up of patients, which they deemed unfeasible once the EMT has left the field. These views are consistent with WHO’s guidance on After action Review, which calls for review within three months after official declaration of the end of the event. While we do not dispute the challenges involved, we think that longer-term follow-up and review, likely collaborating with local healthcare providers, must nevertheless be considered to achieve continuity of care, as well as a full understanding of optimal approaches and the sustainable impact of EMT deployments. Issues of post-deployment follow-up have been increasingly signalled in other research [10, 36].

Participants favoured team-led evaluations, such as by the EMT leader or external consultants. EMT members are expected to undergo competency-based training prior to deploying, so the most appropriate skills are brought to the field, and team leaders are expected to undergo specific leadership training [37], which could enhance their awareness and participation in deployment evaluation. A clear command and communication structure within the EMT can facilitate decision-making [24]. Team members should be well-informed about the deployment context before deployment [38].

For EMTs to be effective, collaboration among key players—such as local governments, host organisations, the WHO, and NGOs—is essential. Each stakeholder influences EMT operations, requiring active engagement from all [39]. Timely information sharing is critical to minimise delays in responding to disaster victims, potentially reducing morbidity and mortality [12]. During sudden onset disasters, EMTs should report to local health ministries to enhance coordination, and a robust reporting system led by the WHO is essential [39, 40]. Additionally, EMTs must respect host country regulations, as accepting international aid can have political implications [41]. Effective EMT deployment requires the host country to request and approve assistance [42]. Life-saving interventions can only be performed within 48 h of a sudden onset disaster, but they often arrive too late [43]. EMTs should have ready-to-deploy equipment and restock supplies locally for better self-sufficiency [14, 44]. Real-time patient data such as epidemiological data and health needs can help identify underserved populations [45], maintain care quality, and for early outbreak detection [46, 47].

EMTs provide crucial primary care when local services are compromised [21, 22]. In areas with weakened local health infrastructure, EMTs may receive patients from clinics or act as referral centres for local hospitals to prevent overwhelming local facilities [48, 49]. Building relationships with local health providers boosts community confidence and facilitates the resumption of local services [47]. As humanitarian assistance evolves, considering the perspectives of patients and communities is essential to align aid with their needs [50, 51]. Sustainability of care post-deployment is increasingly important, particularly for ongoing health needs [20, 52]. Developing follow-up protocols, and longer deployments can enhance sustainability [23].

Current guidelines focus on clinical care but often overlook aspects like patient safety and mental health [53]. Shared challenges include language barriers and cultural differences, which can affect community acceptance [54, 55]. Equipment must be suitable for local conditions [12, 56], and teams must be prepared for unstable logistics [57, 58].

Effective coordination of resources is essential for targeting aid, aligning capabilities with local needs, based on rapid needs assessments after sudden onset disasters [12, 39, 47, 59]. EMTs should also publish all collected data and establish a global information network to share lessons learned with other teams [54].

The recent upheaval in the global health architecture and funding begs the question of whether WHO or another UN-based international organisation is the appropriate coordinating body for EMTs, including for deployment evaluation (62). This question is largely beyond the scope of our study, most of which was conducted before the onset of these radical changes. Nevertheless, regardless of whether WHO or another international body is to ultimately play the primary coordinating role, as long as EMTs exist in some form, the need for evaluation will exist, and thus our research remains pertinent. Furthermore, the scrutiny that our research brings to the EMT landscape may help to assess its true value and appropriate place in the repertoire of global health offerings. In particular, transformation to ensure that the whole EMT process is appropriately community- and local stakeholder-driven is a major question and goal (63). Moreover, in situations where conflict may arise among stakeholders, the presence of a neutral coordinating body such as WHO is crucial to ensure coherence and collective action.

We note the following limitations in our study:

- Participation was below 60%, with potential selection bias due to purposive sampling, and some key stakeholders or regions may be underrepresented, limiting generalizability to all global EMT contexts.
- Although data completeness was high, many participants did not provide rationales for their responses, and the study focused on identifying agreement rather than exploring reasons for disagreement, which may have limited insight into contentious areas.
- Conducting the survey only in English and excluding contexts like armed conflicts may have restricted broader input.
- The proposed framework still requires validation in operational EMT settings.

## Conclusions

This study aimed to develop a consensus-based evaluation framework for EMT deployments using a modified Delphi method. The resulting evaluation themes and questions reflect strong stakeholder consensus and may inform future framework development. The consensus achieved suggests a shared understanding of core domains for assessing EMT deployments, particularly in the area of health operations and technical expertise. Despite a number of limitations, detailed above, the Delphi approach enabled anonymity and broad engagement, yielding perspectives that provide a valuable foundation for the development of a systematic EMT evaluation tool.

While our study does not result in, and nor do we propose here, a finished EMT evaluation tool, it lays a foundation for further discussion and research toward that goal. Next steps will be to synthesize the information gained through our study into a draft EMT deployment evaluation, including refining the language and data collection methods, with clear definitions and numerators/denominators where relevant. This process must entail iterative feedback from EMT practitioners and, importantly, community members who can represent the views of those who would be on the receiving end of deployments. The scope of the evaluation will also have to be adapted for different disaster types and EMT types. The draft evaluation could then first be tested in training and simulation settings, revised as needed, and then progress to field testing after an actual deployment. This would again be followed by broad stakeholder review and appropriate revision, ultimately creating an optimized evidence-based practical and adaptable tool across different disaster contexts and deployment environments, to enhance quality, accountability, and sustainability of emergency health responses worldwide.

## Supplementary Information

Supplementary Material 1

## Data Availability

The datasets used for analysis in this study are available from the corresponding author on reasonable request.

## Abbreviations

EMT: Emergency Medical Team
CREDES: Conducting and Reporting DElphi Studies
WHO: World Health Organization

## References

[1] United Nations Office for Disaster Risk Reduction. Definition: disaster: PreventionWeb; 2023 [Available from: https://www.preventionweb.net/terminology/disaster#:~:text=A%20sudden%2Donset%20disaster%20is,critical%20infrastructure%20failure%2C%20transport%20accident.

[2] WHO EMT Initiative. WHO EMT Extranet. 2024 [Available from: https://extranet.who.int/emt/

[3] Tan YSA, von Schreeb J. Humanitarian assistance and accountability: what are we really talking about? Prehosp Disaster Med. 2015;30(3):264–70. 25783966 10.1017/S1049023X15000254

[4] Abdelmagid N, Checchi F, Garry S, Warsame A. Defining, measuring and interpreting the appropriateness of humanitarian assistance. J Int Humanit Action. 2019;4(1):14.

[5] Bryant C. Evaluation and accountability in emergency relief. In: Ebrahim A, Weisband E, editors. Global accountabilities: Participation, Pluralism, and public ethics. Cambridge: Cambridge University Press; 2007. pp. 168–92.

[6] Jarrett P, Fozdar Y, Abdelmagid N, Checchi F. Healthcare governance during humanitarian responses: a survey of current practice among international humanitarian actors. Confl Health. 2021;15(1):25. 33838690 10.1186/s13031-021-00355-8 PMC8035763

[7] Jafar AJ, Norton I, Lecky F, Redmond AD. A literature review of medical record keeping by foreign medical teams in sudden onset disasters. Prehosp Disaster Med. 2015;30(2):216–22. 25659602 10.1017/S1049023X15000102

[8] Pérouse de Montclos MA. Humanitarian action in developing countries: who evaluates who? Eval Program Plann. 2012;35(1):154–60. 21168212 10.1016/j.evalprogplan.2010.11.005

[9] Dhungana N, Cornish F. Beyond performance and protocols: early responders’ experiences of multiple accountability demands in the response to the 2015 Nepal earthquake. Disasters. 2021;45(1):224–48. 32458589 10.1111/disa.12425

[10] Redmond AD, Mardel S, Taithe B, Calvot T, Gosney J, Duttine A, et al. A qualitative and quantitative study of the surgical and rehabilitation response to the earthquake in Haiti, January 2010. Prehosp Disaster Med. 2011;26(6):449–56. 22469020 10.1017/S1049023X12000088

[11] Peiris S, Buenaventura J, Zagaria N. Is registration of foreign medical teams needed for disaster response? Findings from the response to typhoon Haiyan. West Pac Surveill Response J. 2015;6:29–33. 10.5365/WPSAR.2015.6.2.HYN_014 PMC4710084 26767132

[12] Kuday AD, Özcan T, Çalışkan C, Kınık K. Challenges faced by medical rescue teams during disaster response: A systematic review study. Disaster Med Pub Health Prep. 2023;17:e548. 38058005 10.1017/dmp.2023.217

[13] Gerdin M, Wladis A, von Schreeb J. Foreign field hospitals after the 2010 Haiti earthquake: how good were we? Emerg Med J. 2013;30(1):e8. 22398849 10.1136/emermed-2011-200717

[14] Kwak YH, Shin SD, Kim KS, Kwon WY, Suh GJ. Experience of a Korean disaster medical assistance team in Sri Lanka after the South Asia tsunami. J Korean Med Sci. 2006;21(1):143–50. 16479081 10.3346/jkms.2006.21.1.143 PMC2733963

[15] Warsame A, Murray J, Gimma A, Checchi F. The practice of evaluating epidemic response in humanitarian and low-income settings: a systematic review. BMC Med. 2020;18(1):315. 33138813 10.1186/s12916-020-01767-8 PMC7606030

[16] Warsame A, Blanchet K, Checchi F. Towards systematic evaluation of epidemic responses during humanitarian crises: a scoping review of existing public health evaluation frameworks. BMJ Global Health. 2020;5(1):e002109. 32133177 10.1136/bmjgh-2019-002109 PMC7042582

[17] Amat Camacho N, Karki K, Subedi S, von Schreeb J. International emergency medical teams in the aftermath of the 2015 Nepal earthquake. Prehosp Disaster Med. 2019;34(3):260–4. 31057142 10.1017/S1049023X19004291

[18] von Schreeb J, Riddez L, Samnegård H, Rosling H. Foreign field hospitals in the recent sudden-onset disasters in Iran, Haiti, Indonesia, and Pakistan. Prehosp Disaster Med. 2008;23(2):144–51. discussion 52–53. 18557294 10.1017/s1049023x00005768

[19] Brolin K, Hawajri O, von Schreeb J. Foreign medical teams in the Philippines after typhoon Haiyan 2013 - who were they, when did they arrive and what did they do? PLoS Curr. 2015;7. 10.1371/currents.dis.0cadd59590724486bffe9a0340b3e718 PMC4447417 26064780

[20] Bartolucci AWD, Redmond T. Comparative review on the cost-effectiveness analysis of relief teams’ deployment to sudden-onset disasters. Prehosp Disaster Med. 2019;34(4):415–21. 31298202 10.1017/S1049023X19004540

[21] Abolghasemi H, Radfar MH, Khatami M, Nia MS, Amid A, Briggs SM. International medical response to a natural disaster: lessons learned from the Bam earthquake experience. Prehosp Disaster Med. 2006;21(3):141–7. 16892878 10.1017/s1049023x00003599

[22] de Ville C. Health lessons learned from the recent earthquakes and tsunami in Asia. Prehosp Disaster Med. 2007;22(1):15–21. 17484358 10.1017/s1049023x00004283

[23] Kreiss Y, Merin O, Peleg K, Levy G, Vinker S, Sagi R, et al. Early disaster response in haiti: the Israeli field hospital experience. Ann Intern Med. 2010;153(1):45–8. 20442270 10.7326/0003-4819-153-1-201007060-00253

[24] Aitken P, Leggat PA, Robertson AG, Harley H, Speare R, Leclercq MG. Leadership and use of standards by Australian disaster medical assistance teams: results of a National survey of team members. Prehosp Disaster Med. 2012;27(2):142–7. 22591665 10.1017/S1049023X12000489

[25] Inter-Agency Standing Committee. IASC Strategic Priorities 2022–2023 2022 [Available from: https://interagencystandingcommittee.org/sites/default/files/migrated/2022-01/IASC%20Strategic%20Priorities%20%282022-2023%29.pdf

[26] Inter-Agency Standing Committee. IASC, Strategic Priorities. 2022–2024 2024 [Available from: https://interagencystandingcommittee.org/inter-agency-standing-committee/iasc-strategic-priorities-2022-2024

[27] Yeung T. Evaluating emergency medical team deployments. London, United Kingdom: London School of Hygiene & Tropical Medicine; 2025.

[28] Organisation for Economic Co-operation and Development. Development Results - An Overview of Results Measurement and Management. 2023.

[29] Jünger SPS, Brine J, Radbruch L, Brearley SG. Guidance on conducting and reporting DElphi studies (CREDES) in palliative care: recommendations based on a methodological systematic review. Palliat Med. 2017;31(8):684–706. 28190381 10.1177/0269216317690685

[30] Moher D, Schulz KF, Simera I, Altman DG. Guidance for developers of health research reporting guidelines. PLoS Med. 2010;7(2):e1000217. 20169112 10.1371/journal.pmed.1000217 PMC2821895

[31] Yeung T, Bausch D, Cerga Pashoja A, Schellenberg J. Stakeholder perspectives on evaluating emergency medical teams deployments. Disaster Med Public Health Prepare. 2026;20:e12. 10.1017/dmp.2025.10295 10.1017/dmp.2025.10295 41486514

[32] Kerlinger FN. Foundations of behavioral research. New York: Holt, Rinehart, and Winston, Inc.; 1973.

[33] World Health Organization. Guidance for after action review (‎‎AAR). 2019.

[34] Hsu C-C, Sandford BA. The Delphi technique: making sense of consensus. Volume 12. Research, and Evaluation: Practical Assessment; 2007.

[35] Janis IL. Victims of groupthink: A psychological study of foreign-policy decisions and fiascoes. Oxford, England: Houghton Mifflin; 1972. viii, 277-viii, p.

[36] Nagata T, Yoshida S, Hasegawa M, Ojino M, Murata S, Ishii M. International medical teams of the Japan medical association: A framework for foreign medical teams. Disaster Med Pub Health Prep. 2016;10(1):4–5. 26423514 10.1017/dmp.2015.109

[37] Amat Camacho N, Hughes A, Burkle FJ, Ingrassia P, Ragazzoni L, Redmond A et al. Education and Training of Emergency Medical Teams: Recommendations for a Global Operational Learning Framework. PLOS Currents. 2016;21(ecurrents.dis.292033689209611ad5e4a7a3e61520d0). 10.1371/currents.dis.292033689209611ad5e4a7a3e61520d0 PMC5104687 27917306

[38] Jackson A, Little M. On the ground in Nias in response to an earthquake–an emergency team’s experience. Emerg Med Australas. 2006;18(2):199–202. 16669947 10.1111/j.1742-6723.2006.00829.x

[39] Hamilton ARL, Södergård B, Liverani M. The role of emergency medical teams in disaster response: a summary of the literature. Nat Hazards. 2022;110(3):1417–26.

[40] Kubo T, Salio F, Koido Y. Breakthrough on health data collection in disasters—knowledge arises in Asia spread to the world. In: Chan EYY, Shaw R, editors. Public health and disasters: health emergency and disaster risk management in Asia. Singapore: Springer Singapore; 2020. pp. 299–312.

[41] Takada Y, Otomo Y, Karki KB. Evaluation of emergency medical team coordination following the 2015 Nepal earthquake. Disaster Med Pub Health Prep. 2021;15(3):308–15. 32172718 10.1017/dmp.2019.155

[42] Broby N, Lassetter JH, Williams M, Winters BA. Effective international medical disaster relief: A qualitative descriptive study. Prehosp Disaster Med. 2018;33(2):119–26. 29534767 10.1017/S1049023X18000225

[43] Arbon P. Applying lessons learned to the Haiti earthquake response. Australasian Emerg Nurs J. 2010;13(1):4–6.

[44] Benner P, Stephan J, Renard A, Petitjean F, Larger D, Pons D, et al. Role of the French rescue teams in diquini hospital: Port-au-Prince, January 2010. Prehosp Disaster Med. 2012;27(6):615–9. 22989450 10.1017/S1049023X12001239

[45] Daftary RK, Cruz AT, Reaves EJ, Burkle FM, Christian MD, Fagbuyi DB, et al. Making disaster care count: consensus formulation of measures of effectiveness for natural disaster acute phase medical response. Prehosp Disaster Med. 2014;29(5):461–7. 25226070 10.1017/S1049023X14000922

[46] Yamashita K, Natsukawa T, Kubo T, Kondo H, Koido Y. Vulnerability of pregnant women after a disaster: experiences after the Kumamoto earthquake in Japan. Prehosp Disaster Med. 2019;34(5):569–71. 31466549 10.1017/S1049023X1900476X

[47] Savage E, Christian MD, Smith S, Pannell D. The Canadian armed forces medical response to typhoon Haiyan. Can J Surg. 2015;58(3 Suppl 3):S146–52. 26100775 10.1503/cjs.013514 PMC4467497

[48] Redmond AD, Li J. The UK medical response to the Sichuan earthquake. Emerg Med J. 2011;28(6):516–20. 20817662 10.1136/emj.2009.089920

[49] Sacchetto D, Raviolo M, Lovesio S, Salio F, Hubloue I, Ragazzoni L. Italian field hospital experience in mozambique: report of ordinary activities in an extraordinary context. Prehosp Disaster Med. 2022;37(4):553–7. 35586879 10.1017/S1049023X22000772 PMC9280068

[50] Laperrière H, Evaluations. International agencies and censorship: A field doer’s viewpoint. Evaluation. 2014;20(3):296–310.

[51] WHO Evaluation Office. Guidance note on integrating health equity, gender equality, disability inclusion and human rights in WHO evaluations. Geneva; 2023.

[52] Aloudat T, Khan T. Decolonising humanitarianism or humanitarian aid? PLOS Glob Public Health. 2022;2(4):e0000179. 36962190 10.1371/journal.pgph.0000179 PMC10021363

[53] El-Khani U, Ashrafian H, Rasheed S, Veen H, Darwish A, Nott D, et al. The patient safety practices of emergency medical teams in disaster zones: a systematic analysis. BMJ Glob Health. 2019;4(6):e001889. 31799001 10.1136/bmjgh-2019-001889 PMC6861101

[54] Pearce A, Mark P, Gray N, Curry C. Responding to the boxing day tsunami disaster in Aceh, indonesia: Western and South Australian contributions. Emerg Med Australas. 2006;18(1):86–92. 16454781 10.1111/j.1742-6723.2006.00810.x

[55] Bompangue D, Oyugi B, Bokulu M, Tshijuke SM, Das T, Conteh IN, et al. COVID-19 as an accelerator of the implementation of emergency medical teams initiative in the AFRO region. Disaster Med Pub Health Prep. 2023;17:e489. 37702057 10.1017/dmp.2023.31

[56] Chauhan A, Chopra BK. Deployment of medical relief teams of the Indian army in the aftermath of the Nepal earthquake: lessons learned. Disaster Med Pub Health Prep. 2017;11(3):394–8. 28031077 10.1017/dmp.2016.146

[57] Redmond AD, Watson S, Nightingale P. The South Manchester accident rescue team and the earthquake in Iran, June 1990. BMJ. 1991;302(6791):1521–3. 1855026 10.1136/bmj.302.6791.1521 PMC1670205

[58] Roshchin GG, Mazurenko OV. Ukranian’s disaster medicine team mission to India following the earthquake of 2001. Prehosp Disaster Med. 2002;17(3):163–6. 12627920 10.1017/s1049023x0000039x

[59] Djalali A, Ingrassia PL, Corte FD, Foletti M, Gallardo AR, Ragazzoni L, et al. Identifying deficiencies in National and foreign medical team responses through expert opinion surveys: implications for education and training. Prehosp Disaster Med. 2014;29(4):364–8. 24945852 10.1017/S1049023X14000600

