# Supplementary material for: Consensus on key domains for emergency medical teams deployment evaluation: a Delphi method study
Source: Confl Health. 2026 Feb 5;20:16. doi: 10.1186/s13031-026-00751-y (PMC12927219; doi:10.1186/s13031-026-00751-y)
Supplement: Supplementary file 1 — Supplementary Material 1 [file 13031_2026_751_MOESM1_ESM.docx]

Appendix 1: Evaluation Themes and Questions and Their Level of Agreement

The percentages that are in red signifies those that did not reach consensus (Agreement Intensity <75%), and did not agree on changes.

Percentages may not sum up to 100% due to rounding.

| **AAR Pillar** | **Round 1** | **Round 2** | **Round 1** **results** | **Round 2 results** | | | | | Notes |
| --- | --- | --- | --- | --- | --- | --- | --- | --- | --- |
|  | **Questions** | **Round 2 changes & new suggestions from Round 1 *Changes are in brown *Questions have been placed in their new evaluation themes if changes have been agreed** | **Suitable** | **Suitable** | **I prefer the original** | **I agree with the change** |  |  |  |
| 1 Leadership | (a) Leadership |  | **100%** |  |  |  |  |  |  |
|  | Was there clear leadership structure for your EMT in the field? |  | **100%** |  |  |  |  |  |  |
|  | Was the management team able to play a defining role in supporting the EMT members in reaching overall goals? |  | **80%** |  |  |  |  |  |  |
|  | Was the management team able to provide confidence to the team members to cope with changing and uncertain situations? |  | **74%** | **94%** |  |  |  |  |  |
|  | Was the management team able to engage with the team for feedback when dealing with changing and uncertain situations? |  | **87%** |  |  |  |  |  |  |
|  | Was the team leader able to ensure coordination within the team to contribute to reaching overall goals? | Move from "Teamwork" to "Leadership" | **93%** |  | 25% | 75% |  |  |  |
|  | Was the team leader able to resolve conflicts within the team, if any occurred? | Move from "Teamwork" to "Leadership" | **100%** |  | 19% | 81% |  |  |  |
|  |  | Was the management team able to set and communicate clear goals, defining roles and responsibilities for each team members? |  | **100%** |  |  |  |  |  |
|  |  | What was the past deployment experience of the management team? |  | **81%** |  |  |  |  |  |
|  |  | Was the management team transparent in their communication? |  | **94%** |  |  |  |  |  |
|  |  | Did the management team establish systems for performance monitoring to provide constructive feedback? |  | **100%** |  |  |  |  |  |
|  | (b) Teamwork |  | **100%** |  |  |  |  |  |  |
|  | Were there defined roles for team members? |  | **93%** |  |  |  |  |  |  |
|  |  | Did the management team foster a positive and cohesive environment among team members? |  | **94%** |  |  |  |  |  |
|  |  | What formal or informal channels were established by the management team for conflict presentation and resolution within the team? |  | **94%** |  |  |  |  |  |
|  |  | When difficulties presented, was there openness to discuss methods on how to resolve them? |  | **100%** |  |  |  |  |  |
|  |  | Did the team members have previous acquaintance in training or did they meet for the first time in this deployment? |  | **82%** |  |  |  |  |  |
| 2. Partner Coordination | (c) Deploying organisation |  | **100%** |  |  |  |  |  |  |
|  | Did the coordination with the deploying organisation ensure smooth departure from the EMT's home country? |  | **86%** |  |  |  |  |  |  |
|  | Was the coordination with the deploying organisation effective in reaching overall goals? |  | **93%** |  |  |  |  |  |  |
|  |  | Was the deploying organisation supportive when difficulties presented during the deployment? |  | **100%** |  |  |  |  |  |
|  | (d) Local communities |  | **93%** |  |  |  |  |  |  |
|  | Were there any cultural challenges that had an impact on the effectiveness of the deployment? |  | **93%** |  |  |  |  |  |  |
|  | Were there any channels for patients to provide feedback to your EMT in relation to the services provided by your EMT? | What were the formal or informal channels for patients to provide feedback to your EMT in relation to the services provided by your EMT? | **86%** |  | 13% | 88% |  |  |  |
|  | Was the leader of your EMT able to ensure coordination between your EMT and different partners in the field in reaching overall goals? |  | **86%** |  |  |  |  |  |  |
|  | Were there any channels for partner organisations to provide feedback to your EMT in relation to the services provided by your EMT? | What were the formal or informal channels for local communities or organisations to provide feedback to your EMT in relation to the services provided by your EMT? | **87%** |  | 19% | 81% |  |  |  |
|  |  | What kinds of local communities or organisations have the opportunity to provide feedback? |  | **87%** |  |  |  |  |  |
|  |  | Does the EMT have clear processes for responding to and acting upon feedback received? |  | **100%** |  |  |  |  |  |
|  |  | Are the results of all feedback received shared openly? |  | **94%** |  |  |  |  |  |
|  | (e) Host organisations | Local government / Ministry of Health | **100%** |  | 31% | 69% |  |  | Disagreed changes |
|  | Did host organisations establish channels to share knowledge with the EMT? | What were the formal or informal channels established by the local government or Ministry of Health to share knowledge with the EMT?  Move from "Partner Coordination with Host organisations" to "Information management" | **93%** |  | 19% | I agree with both the change in text and the move 31% | I agree with the change in text only  38% | I agree with the move only  13% | Disagreed changes |
|  | Were there any channels for host organisations to provide feedback to your EMT in relation to the services provided by your EMT? | What were the formal or informal channels for the local government / Ministry of Health to provide feedback to your EMT in relation to the services provided by your EMT? | **74%** | **94%** |  |  |  |  |  |
|  |  | Was the coordination between the local government / Ministry of Health and the WHO effective in reaching overall goals? |  | **100%** |  |  |  |  |  |
|  |  | What type of support did your EMT receive from the local government / Ministry of Health? |  | **100%** |  |  |  |  |  |
|  | (f) WHO, EMT CC and other EMTs |  | **100%** |  |  |  |  |  |  |
|  | Did the WHO establish channels to share knowledge in the field? | What were the formal or informal channels established by WHO to share knowledge in the field? | **80%** |  | 13% | 88% |  |  |  |
|  | Did the WHO establish channels to share data in the field? | What were the formal or informal channels established by WHO to share data in the field? | **80%** |  | 13% | 88% |  |  |  |
|  | Was WHO's coordination among various EMTs and other stakeholders in the field effective in reaching overall goals?? |  | **80%** |  |  |  |  |  |  |
|  | Did the WHO facilitate collaboration among different EMTs and other stakeholders in the field? |  | **80%** |  |  |  |  |  |  |
|  | How often were the WHO EMT Coordination Cell management meetings held throughout your EMT's deployment? |  | **86%** |  |  |  |  |  |  |
|  |  | Was the frequency of EMT Coordination Cell management meetings adequate in reaching overall goals? |  | **100%** |  |  |  |  |  |
|  |  | Was your deployment coordinated through the WHO EMT Coordination Cell in the affected country? |  | **100%** |  |  |  |  |  |
|  |  | Was the overall coordination of the Office for the Coordination of Humanitarian Affairs (OCHA) and the Health Cluster with other stakeholders in the field effective in reaching overall goals? |  | **100%** |  |  |  |  |  |
|  |  | Was the coordination with United Nations Disaster Assessment and Coordination (UNDAC) effective in reaching overall goals? |  | **100%** |  |  |  |  |  |
|  |  | Did you receive a WHO EMT Coordination Cell monitoring visit? If so, how did the visit contribute to the EMT performance? |  | **100%** |  |  |  |  |  |
|  |  | What type of support did your EMT receive from WHO or the EMT Coordination Cell? |  | **100%** |  |  |  |  |  |
| 3. Information management and planning | (g) Preparation of evaluation | Move from "Information management and planning" to "Operations support and logistics" | **93%** |  | 46% | 54% |  |  | Disagreed changes |
|  | Was an evaluation/review of the deployment planned prior to deployment? | Move from "Information management and planning" to "Operations support and logistics" | **74%** | **88%** | 46% | 54% |  |  | Disagreed changes |
|  | Were there dedicated personnel in charge of the evaluation / review? | Move from "Information management and planning" to "Operations support and logistics" | **80%** |  | 46% | 54% |  |  | Disagreed changes |
|  |  | (h) Information management |  | **94%** |  |  |  |  |  |
|  | Did host organisations establish channels to share data with the EMT? | What were the formal or informal channels established by the local government or Ministry of Health to share data with the EMT? Move from "Partner Coordination with Host organisations" to "Information management" | **73%** | **88%** |  |  |  |  |  |
|  |  | What kind of knowledge or data was shared by the WHO? |  | **94%** |  |  |  |  |  |
|  |  | What kind of knowledge or data was shared by local government / Ministry of Health? |  | **100%** |  |  |  |  |  |
|  |  | Did your EMT report to the EMTCC or the local government / Ministry of Health? |  | **100%** |  |  |  |  |  |
|  |  | How often did your EMT report to the EMTCC or the local government / Ministry of Health? |  | **94%** |  |  |  |  |  |
|  |  | What kind of knowledge or data did you report to the EMTCC or the local government / Ministry of Health? |  | **100%** |  |  |  |  |  |
|  |  | Did any other stakeholders share any knowledge or data? How were they shared? |  | **88%** |  |  |  |  |  |
|  | (i) Documentation | Move from "Operations support and logistics" to "Information management and planning" | **100%** |  | 0% | 100% |  |  |  |
|  | During the deployment, did the team hold daily briefings? |  | **100%** |  |  |  |  |  |  |
|  | Were challenges and how they were resolved well documented? | Move from "Operations support and logistics" to "Information management and planning" | **87%** |  | 0% | 100% |  |  |  |
|  | Did the EMT comply with the requirement of daily submission of Minimum Data Set (MDS)? | Move from "Operations support and logistics" to "Information management and planning" | **100%** |  | 0% | 100% |  |  |  |
|  |  | Was the Minimum Data Set (MDS) used to send reports to the EMTCC? |  | **100%** |  |  |  |  |  |
|  |  | Was documentation mainly computerised or was it on paper? |  | **82%** |  |  |  |  |  |
|  |  | Was patient data recorded for future research? |  | **75%** |  |  |  |  |  |
|  |  | How was patient data collected in the field? |  | **94%** |  |  |  |  |  |
| 4. Health operations and technical expertise | (j) Team members' experience |  | **100%** |  |  |  |  |  |  |
|  | How many people were deployed in the EMT for the whole deployment? |  | **93%** |  |  |  |  |  |  |
|  | Did all team members have appropriate practice certification? |  | **87%** |  |  |  |  |  |  |
|  | Regardless of their technical expertise, were the deployed team members suitable for the deployment? |  | **80%** |  |  |  |  |  |  |
|  | Did the trainings and pre-deployment activities prepare members sufficiently for the deployment? |  | **86%** |  |  |  |  |  |  |
|  | After the deployment, did team members have the opportunity to discuss the technical aspects of the deployment? |  | **86%** |  |  |  |  |  |  |
|  | After the deployment, did team members have the opportunity to talk about the non-technical aspects of the deployment? |  | **86%** |  |  |  |  |  |  |
|  |  | Was the procedure for getting a license to practice complicated? |  | **88%** |  |  |  |  |  |
|  |  | How many team members were deployed for each role? |  | **100%** |  |  |  |  |  |
|  |  | What pre-deployment trainings have team members been through? |  | **94%** |  |  |  |  |  |
|  |  | Was there just in time training provided to team members prior to deployment? |  | **100%** |  |  |  |  |  |
|  |  | What was the ratio between first deployment and those with experience amongst all team members? |  | **100%** |  |  |  |  |  |
|  |  | How many team members worked in their home setting? |  | **88%** |  |  |  |  |  |
|  |  | Was there task shifting during the deployment? |  | **94%** |  |  |  |  |  |
|  |  | Were there team members who did not complete their full duty and if so, why? |  | **88%** |  |  |  |  |  |
|  |  | Was there mental health support for staff during and after the deployment? |  | **100%** |  |  |  |  |  |
|  | (k) Filling in gaps of needs | Move from "Information management and planning" to "Health operations and technical expertise" | **86%** |  | 25% | 75% |  |  |  |
|  | Was there any gap between pre-deployment briefing and reality that affected operational ability in the field? | Move from "Information management and planning" to "Health operations and technical expertise" | **94%** |  | 25% | 75% |  |  |  |
|  | Was the local community or government consulted regarding to health needs? | Move from "Information management and planning" to "Health operations and technical expertise" | **100%** |  | 25% | 75% |  |  |  |
|  | Were the services offered by the EMT relevant to the health needs of the communities? | Move from "Information management and planning" to "Health operations and technical expertise" | **94%** |  | 25% | 75% |  |  |  |
|  | Was there continuous assessment of the health needs in the location your EMT worked in? | Move from "Information management and planning" to "Health operations and technical expertise" | **93%** |  | 25% | 75% |  |  |  |
|  | Was the geographical location of the field hospital or clinic appropriate for serving communities with health needs? | Move from "Information management and planning" to "Health operations and technical expertise" | **94%** |  | 25% | 75% |  |  |  |
|  | Did the EMT deploy quickly enough to support with the direct consequences of the event? | Move from "Information management and planning" to "Health operations and technical expertise" | **73%** | **87%** | 25% | 75% |  |  |  |
|  | Did the EMT support the management of coincidental emergencies that were being managed by the local healthcare system? | Move from "Information management and planning" to "Health operations and technical expertise" | **80%** |  | 25% | 75% |  |  |  |
|  | Did the EMT support the management of chronic conditions that were being managed by the local healthcare system? | Move from "Information management and planning" to "Health operations and technical expertise" | **73%** | **82%** | 25% | 75% |  |  |  |
|  |  | Were the health needs of the population received by your EMT similar to those identified from the needs assessment? |  | **94%** |  |  |  |  |  |
|  |  | Was the EMT re-tasked or changed location due to changing needs? |  | **87%** |  |  |  |  |  |
|  | (l) Patient Numbers |  | **100%** |  |  |  |  |  |  |
|  | How many patients were treated per day? |  | **94%** |  |  |  |  |  |  |
|  | Were the patients triaged so there can be effective use of resources available? |  | **100%** |  |  |  |  |  |  |
|  | How many consultations were provided per day? |  | **100%** |  |  |  |  |  |  |
|  | How many specialist consultations were provided per day? |  | **94%** |  |  |  |  |  |  |
|  | How many patients were discharged per day? |  | **80%** |  |  |  |  |  |  |
|  | How long were patients' length of stay? |  | **87%** |  |  |  |  |  |  |
|  | What was the mortality rate for the whole deployment? |  | **100%** |  |  |  |  |  |  |
|  | What was the hospital occupancy rate for the whole deployment? |  | **93%** |  |  |  |  |  |  |
|  | How long did it take for patients to arrive at the hospital? |  | **67%** | **69%** |  |  |  |  |  |
|  | Did the patient numbers meet with the EMT's expectations? |  | **80%** |  |  |  |  |  |  |
|  |  | How many inpatient admissions were there per day? |  | **100%** |  |  |  |  |  |
|  |  | How many outpatient consultations were there per day? |  | **100%** |  |  |  |  |  |
|  |  | What was the main causes of mortality? |  | **100%** |  |  |  |  |  |
|  |  | How many patients had repeat visits and were these counted as new patients? |  | **88%** |  |  |  |  |  |
|  |  | What was the proportion of disaster-related cases among all caseload? |  | **100%** |  |  |  |  |  |
|  |  | What was the proportion of non disaster-related, underlying non-communicable diseases among all caseload? |  | **100%** |  |  |  |  |  |
|  |  | What was the proportion of non disaster-related communicable diseases among all caseload? |  | **100%** |  |  |  |  |  |
|  |  | What was the proportion of non disaster-related maternal and child health cases among all caseload? |  | **100%** |  |  |  |  |  |
|  | (m) Clinical Care |  | **100%** |  |  |  |  |  |  |
|  | How many of each type of medical condition was diagnosed per day? |  | **100%** |  |  |  |  |  |  |
|  | How many of each test was requested per day? |  | **87%** |  |  |  |  |  |  |
|  | How were the treatments followed through? |  | **74%** | **75%** |  |  |  |  |  |
|  |  | Were there significant differences between EMTs SOPs and national procedures/treatments? How was that solved? |  | **94%** |  |  |  |  |  |
|  | How were prescriptions given? |  | **93%** |  |  |  |  |  |  |
|  | How many of each type of surgery was performed per day? |  | **100%** |  |  |  |  |  |  |
|  | What was the amputation rate? |  | **87%** |  |  |  |  |  |  |
|  | How many babies were delivered per day? |  | **100%** |  |  |  |  |  |  |
|  |  | How many patients had more than one procedure done? |  | **94%** |  |  |  |  |  |
|  |  | How many surgical interventions were done per inpatient? |  | **94%** |  |  |  |  |  |
|  |  | How much of each type of anaesthesia or sedation was used? |  | **82%** |  |  |  |  |  |
|  | (n) Referral of Patients |  | **100%** |  |  |  |  |  |  |
|  | Were the referral mechanisms to and from different health entities smooth? |  | **100%** |  |  |  |  |  |  |
|  | Were there staff and transportation available for all transfers? |  | **87%** |  |  |  |  |  |  |
|  | Did the EMT use WHO's patient referral forms or other standardised forms when referring patients? |  | **87%** |  |  |  |  |  |  |
|  | How many patients were referred to the EMT by other clinics or hospitals? |  | **100%** |  |  |  |  |  |  |
|  | How many patients were referred from the EMT to other hospitals? |  | **100%** |  |  |  |  |  |  |
|  | Were local rehabilitation services referred to patients that need it? | Move from "Clinical Care" to "Referral of patients" | **87%** |  | 13% | 88% |  |  |  |
|  |  | What were the indications for referral? |  | **88%** |  |  |  |  |  |
|  |  | How many patients were referred to a higher level health facility? |  | **100%** |  |  |  |  |  |
|  |  | How many patients were referred to a lower level health facility? |  | **81%** |  |  |  |  |  |
|  |  | How many patients were referred to treatment outside the disaster zone? |  | **94%** |  |  |  |  |  |
|  |  | How many patients were referred for treatment in other countries? |  | **100%** |  |  |  |  |  |
|  |  | Were any patients sent to the EMT’s home country for treatment? |  | **75%** |  |  |  |  |  |
|  |  | For how long were patients followed up after they were referred somewhere else? |  | **75%** |  |  |  |  |  |
|  | (o) Local engagement |  | **100%** |  |  |  |  |  |  |
|  | Did the EMT conduct any community health education? |  | **93%** |  |  |  |  |  |  |
|  | Was the EMT set up as a field hospital or was it part of an existing local facility? |  | **87%** |  |  |  |  |  |  |
|  | Did the EMT support the improvement of local emergency capacity? | Did the EMT support the improvement of local emergency capacity, such as conducting trainings or education for local staff? | **87%** |  | 25% | 75% |  |  |  |
|  |  | How were the EMT services advertised among the community? |  | **88%** |  |  |  |  |  |
|  |  | Did you conduct community assessments? |  | **100%** |  |  |  |  |  |
|  | (p) Post-deployment follow-up |  | **100%** |  |  |  |  |  |  |
|  | Did the EMT do any follow-up to patients after the it exited the country? | Either to keep the original and add two new questions OR just having the two new questions. | **60%** | **57%** | I prefer just having the two new questions  56% | I prefer keeping the original & adding two new questions  44% |  |  |  |
|  |  | For how long were patients followed up after they were discharged? |  | **88%** |  |  |  |  |  |
|  |  | Did the EMT continue contact with local communities or local government or Ministry of Health after they exited the country? |  | **100%** |  |  |  |  |  |
|  |  | Were there repeat visits of EMT members to the disaster area after the deployment? |  | **69%** |  |  |  |  |  |
| 5. Operations support and logistics | (q) Arrival |  | **100%** |  |  |  |  |  |  |
|  | How many days passed between the onset of disaster and the EMT arriving? |  | **100%** |  |  |  |  |  |  |
|  | Was the arrival into the host country smooth? |  | **93%** |  |  |  |  |  |  |
|  |  | Who solicited and agreed to your EMT's deployment? |  | **88%** |  |  |  |  |  |
|  |  | Did your EMT register with the local government or with the EMTCC upon arrival? |  | **94%** |  |  |  |  |  |
|  |  | Did your EMT experience any difficulties related to your arrival that impacted your ability to start working effectively in the host country? |  | **94%** |  |  |  |  |  |
|  |  | How many days passed between your EMT's arrival into host country and the first operational day? |  | **94%** |  |  |  |  |  |
|  | (r) Quality Assurance |  | **100%** |  |  |  |  |  |  |
|  | Did the team provide the services and expertise they pledged? |  | **93%** |  |  |  |  |  |  |
|  | Was the EMT self-sufficient in accordance with the minimum standards? |  | **94%** |  |  |  |  |  |  |
|  | How well did the EMT comply with their SOPs? |  | **100%** |  |  |  |  |  |  |
|  | Were morbidity & mortality reviews done by the EMT? | Move from "Documentation" to "Quality assurance" | **93%** |  | 19% | 81% |  |  |  |
|  |  | Did your EMT alter the SOPs to match local needs and context? |  | **94%** |  |  |  |  |  |
|  | (s) Equipment, supplies and logistics |  | **94%** |  |  |  |  |  |  |
|  | Was the team member's personal equipment sufficient for the needs of the deployment? |  | **93%** |  |  |  |  |  |  |
|  | Was the team equipment brought appropriate for the needs of the deployment? |  | **94%** |  |  |  |  |  |  |
|  | Were the medical tangible resources sufficient for the needs of the deployment? |  | **94%** |  |  |  |  |  |  |
|  | Was there sufficient water supply for the EMT to maintain its operations? |  | **100%** |  |  |  |  |  |  |
|  | Did the EMT donate any equipment or medication when they exited? |  | **94%** |  |  |  |  |  |  |
|  |  | Was any equipment procured locally? |  | **94%** |  |  |  |  |  |
|  |  | Was there a system in place to resupply equipment during the deployment? |  | **94%** |  |  |  |  |  |
|  |  | Were there any difficulties related to your EMT's self-sufficiency? |  | **100%** |  |  |  |  |  |
|  |  | Did the host country or WHO provided any logistical support to your EMT? |  | **100%** |  |  |  |  |  |
|  |  | (t) Safety |  | **94%** |  |  |  |  |  |
|  |  | Did any safety and security issues arise before, during and after the deployment? |  | **100%** |  |  |  |  |  |
|  | (u) Exit |  | **100%** |  |  |  |  |  |  |
|  | Did the EMT continuously review the exit strategy throughout the deployment? |  | **93%** |  |  |  |  |  |  |
|  | Did the EMT fill in and submit the EMT exit report form to host organisations or WHO? |  | **93%** |  |  |  |  |  |  |
|  | How long did the EMT stay for? |  | **93%** |  |  |  |  |  |  |
| 6. Finance and administration | (v) Finance & Administration |  | **100%** |  |  |  |  |  |  |
|  | How much did the whole deployment cost? |  | **93%** |  |  |  |  |  |  |
|  | Was the EMT's administrative support sufficient in the field? |  | **87%** |  |  |  |  |  |  |
|  |  | How much does each component of the deployment cost? |  | **88%** |  |  |  |  |  |
|  |  | Were there any difficulties during the deployment due to financial considerations? |  | **88%** |  |  |  |  |  |
|  | (w) Publications and reports |  | **100%** |  |  |  |  |  |  |
|  | Did the EMT compile a report documenting the whole deployment? |  | **93%** |  |  |  |  |  |  |
|  | Did the EMT publish in any scientific journals after the deployment? | Add new question (below) | **74%** | **82%** |  |  |  |  |  |
|  |  | Did the EMT publish anything publicly available? |  | **94%** |  |  |  |  |  |
|  | Did the EMT include lessons learned in any publicly available report or publication |  | **94%** |  |  |  |  |  |  |
|  |  | Was there any media engagement before, during or after the deployment? |  | **94%** |  |  |  |  |  |
